# Is there an Increased Risk for Unfavorable Obstetric Outcomes in Women with Endometriosis? An Evaluation of Evidences

**DOI:** 10.1055/s-0040-1708885

**Published:** 2020-04

**Authors:** Giuliana Annicchino, Helena Malvezzi, Carla de Azevedo Piccinato, Sérgio Podgaec

**Affiliations:** 1 Hospital Israelita Albert Einstein, São Paulo, SP, Brazil

**Keywords:** endometriosis, pregnancy complications, obstetric complications, pregnancy outcomes, obstetric outcomes, endometriose, complicações da gravidez, complicações obstétricas, desfechos da gravidez, desfechos obstétricos

## Abstract

**Objective** The present study is a systematic review of the literature to assess whether the presence of endometriosis determines or contributes to adverse obstetric outcomes.

**Data Sources** The present work was carried out at the Hospital Israelita Albert Einstein, São Paulo, state of São Paulo, Brazil, in accordance to the PRISMA methodology for systematic reviews. A review of the literature was performed using PubMed, Web of Science and Scopus databases. The keywords used were: *pregnancy* *outcome*, *pregnancy* *complications*, *obstetrical* *complications*, *obstetrics*, *obstetric* *outcomes* and *endometriosis*. The survey was further completed by a manually executed review of cross-referenced articles, which was last performed on November 30, 2018.

**Selection of studies** The survey disclosed a total of 2,468 articles, published from May 1946 to October 2017. A total of 18 studies were selected to be further classified according to their quality and relevance.

**Data Collection** The Newcastle–Ottawa Quality Assessment Scale was used for classification. Five studies of greater impact and superior evidence quality and 13 studies of moderate evidence quality were selected. We analyzed the studies for the characteristics of their patients plus how endometriosis was diagnosed and their respective obstetric outcomes taking into account their statistical relevance.

**Data Synthesis** Analyses of the higher impact and better quality studies have shown high incidence of preterm birth and placenta previa in patients with endometriosis.

**Conclusion** Placenta previa and preterm birth are the most statistically significant outcomes related to endometriosis, as indicated by our systematic review. The present information is useful to alert obstetricians and patients about possible unfavorable obstetric outcomes.

## Resumo

**Objetivo** Realizar uma revisão sistemática e crítica da literatura de modo a avaliar se a presença de endometriose determina desfechos obstétricos adversos na gestação.

**Fonte dos dados** O presente estudo foi realizado no Hospital Israelita Albert Einstein, São Paulo, SP, Brasil, de acordo com a metodologia PRISMA para revisões sistemáticas. As bases de dados usadas para a revisão de literatura foram Pubmed, Web of Science e Scopus. As palavras-chave usadas foram: *pregnancy* *outcome*, *pregnancy* *complications*, *obstetrical* *complications*, *obstetrics*, *obstetric* *outcomes* e *endometriosis*. Uma revisão manual de artigos com referências cruzadas completou a pesquisa, que foi realizada pela última vez em 30 de novembro de 2018.

**Seleção dos estudos** A pesquisa contou com o total de 2.468 artigos, publicados de maio de 1946 a outubro de 2017. Foram selecionados 18 estudos com base em sua relevância.

**Coleta de dados** A metodologia Newcastle–Ottawa Quality Assessment Scale foi usada para selecionar 5 estudos cuja evidência era de melhor qualidade e 13 estudos de moderada qualidade de evidência. As características das populações dos estudos foram analisadas, assim como a doença endometriose foi diagnosticada e os respectivos desfechos obstétricos nas pacientes observando-se a relevância estatística dos estudos.

**Síntese dos dados** A análise dos estudos de maior impacto e de melhor qualidade de evidência mostram que placenta prévia e ocorrência de nascimentos pré-termo são os desfechos obstétricos desfavoráveis de maior incidência em pacientes com endometriose.

**Conclusão** Placenta prévia e nascimentos pré-termo são os desfechos obstétricos com maior significância estatística relacionados à endometriose. Esta informação é útil para alertar obstetras e pacientes com endometriose para possíveis desfechos obstétricos desfavoráveis.

## Introduction

Endometriosis is defined by the presence of endometrial (glandular and/or stromal) tissue outside the uterus. The most frequent sites of lesion are the pelvic viscera and the peritoneum, and the disease can be classified as superficial, deep or ovarian and/or peritoneal. The most severe forms can lead to deformities of the Fallopian ducts and may affect the urinary tract and intestinal walls.^1^ It is estimated that endometriosis affects 10% of women of reproductive age, is associated with pelvic pain in 30% and causes infertility in 30 to 40%.^2^ ^3^ ^4^ In recent years, there has been considerable progress in understanding the pathogenesis, the evolution, the diagnosis and the treatment of the disease.^5^

It is important to emphasize that infertility alone is already associated to a greater risk of obstetric complications such as pre-eclampsia, gestational hypertension, prematurity, hemorrhage before delivery and the need of cesarean section.^6^ Some studies postulated the association of endometriosis with unfavorable obstetric outcomes, such as pre-eclampsia or spontaneous hemoperitoneum, and the occurrence of sigmoid perforation or appendicitis.^7^ ^8^ While not yet clarified, these associations may occur due to endometrial resistance to progesterone, inadequate uterine contractions, excessive stimulation of the endometrium caused by free radicals, changes in the uterine junctional zone, and inflammatory processes causing endometrial, peritoneal and systemic manifestations.^1^ ^9^ ^10^ ^11^ ^12^ These mechanisms will be addressed in the discussion of the present study.

Two systematic reviews that related endometriosis to gestational risks have been recently published; however, there is a methodological gap regarding the heterogeneity among the groups studied, the confirmatory diagnosis of endometriosis, the sample size of each published study, and the inclusion of patients who were already classified as having high-risk gestations (symptomatic patients who sought out clinics and hospitals).^13^ ^14^

Therefore, the goal of our study was to perform a systematic review of the literature to assess whether the presence of endometriosis in fact results in adverse obstetric outcomes. We took the data quality of the analyzed articles into consideration to reach the conclusions.

## Methods

The present study was carried out at the Hospital Israelita Albert Einstein, São Paulo, state of São Paulo, Brazil, according to the PRISMA methodology for systematic reviews. To identify relevant articles to be included in the study, a review of the literature was done using the PubMed, Web of Science and Scopus databases. The keywords used were: *pregnancy* *outcome*, *pregnancy* *complications*, *obstetrical* *complications*, *obstetrics*, *obstetric* *outcomes* and *endometriosis.* The search period was from May 1946 to October 2017. A manual review of cross-referenced articles completed the survey, which was last performed on August 30, 2018.

## Studies Selection

Studies were selected using the following predetermined inclusion criteria: [i]women who had a diagnosis of endometriosis during or before pregnancy compared to a control group of women without the diagnosis [ii] any outcomes of interest in the present pregnancy, and [iii] observational, cohort or case-controlled human study design that were reported in English. The primary outcomes of the present study were determined previously and included the following adverse obstetric and perinatal outcomes: abortion, ectopic gestation, fetal loss, pre-eclampsia, bleeding during pregnancy, placental retention, placenta previa, premature placental abruption, premature membranes rupture, preterm labor, cesarean section, postpartum hemorrhage, preterm delivery, small for gestational age (SGA) fetus, stillborn neonate and neonatal death. The secondary outcomes were the presence of any other clinically important adverse pregnancy outcomes reported in the literature. Information extracted from each study included: the country where the research was done; the name of the cohort study; duration and sample size; inclusion criteria; definition of reference or control group; endometriosis diagnostic criteria; obstetric or neonatal outcomes; demographics to which the studies were adjusted.

## Selection Criteria

We excluded from the analyses the studies that were not prospective or retrospective cohort or case-control, as well as those not written in English or lacking data. The study selection process, full text screening, and data extraction was conducted independently by two researchers (Annicchino G. and Piccinato C. A.), following the PRISMA guidelines. Disagreements were solved after consulting a third opinion (Podgaec S.).

## Data Extraction

One review author (Annicchino G.) independently standardized the data extraction approach from the eligible studies. Information was gathered on the cohort configuration, endometriosis diagnosis and its stage, conceptive method, use of assisted reproductive techniques and detailed obstetric and perinatal outcomes.

## Data Analysis

Data for adverse outcomes were collected as dichotomous data, and the results are presented as odds ratios (ORs) with 95% confidence interval (CI).

## Assessment of Bias Risk

The quality of the included studies was assessed by the Newcastle-Ottawa Quality Assessment Scale (NOS, scores of 0–9 stars) for the selection of study groups (up to 4 stars/points); comparability of groups (up to 2 stars/points); and, the ascertainment of either the exposure or outcome of interest for case-control or cohort studies, respectively (up to 3 stars/points).^15^

## Results

**Fig. 1:**
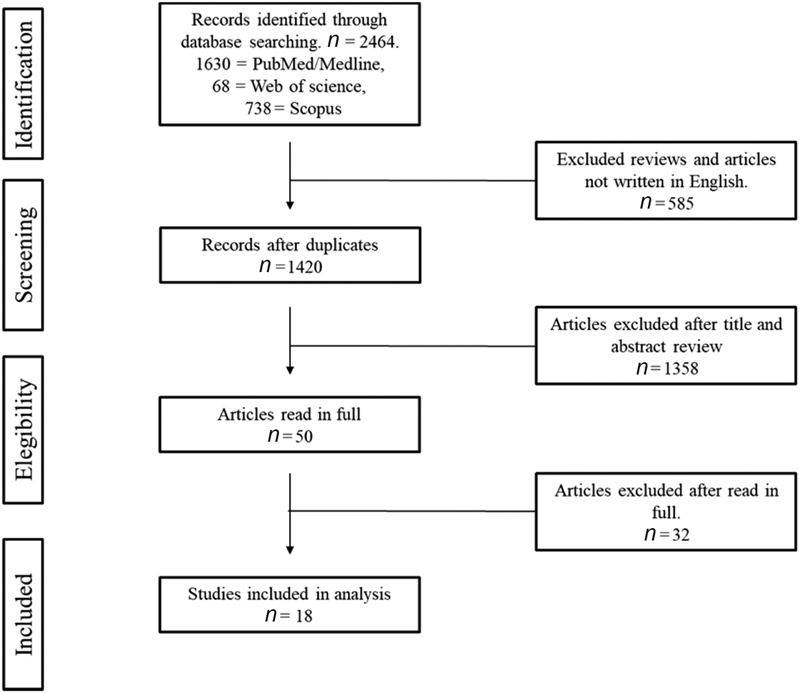
Flowchart of the method used to select the articles (Prisma).

The search identified a total of 2,468 articles, ranging from May 1946 to October 2017, of which 1,630 were from PubMed, 738 from Scopus and 68 from Web of science, 459 duplicates and 585 revisions or published in other languages. By limiting the research to only English-written articles, and excluding duplicate articles and systematic reviews, we found 1,358 articles. The initial selection was done by reading the titles and abstracts of the articles. All case-control, prospective or retrospective cohort studies evaluating obstetric outcomes in women diagnosed with endometriosis were included. Fifty articles were read in full. No date limit was imposed, and two reviewers (Annicchino G. and Podgaec S.) independently nonblindly assessed the eligibility of the articles following the standardized protocol. Disagreement regarding the inclusion of studies were discussed and, by consensus, the articles were included or excluded. The references of these articles were also searched resulting in finding one additional study. A systematic review was performed analyzing the year of publication, number of patients involved in the study, type of study, and the results and conclusions of each study resulting in the selection of 18 studies (Fig. 1).

All of the articles within the described theme were included, regardless of the age of the patients, type of pregnancy (single or multiple), gestational age or form of conception (natural or artificial). The diagnosis method of endometriosis was not taken into consideration for exclusion or inclusion purposes; it could be clinical, surgical or histopathological. These 18 studies were classified according to their relevance using the NOS scale (scores of 0–9 stars). Studies with NOS ≥ 4 were regarded as moderate quality and ≥ 8 were regarded as high-quality. According to this evaluation, 5 studies of greater impact and quality of superior evidence and 13 studies of moderate quality of evidence were selected (Supplementary Material Appendix 1). Table 1 exhibits the data of the control groups, how the disease was diagnosed, and other particularities of the studies. Tables 2a, 2b and 2c display the studies in which the obstetric outcomes were studied in relation to endometriosis and their statistical relevance.

**Table 1:** Study population and methodology of the evaluated studies according to the diagnosis of endometriosis, conceptive method, and criteria of analyses (FIV and endometriosis stage)

Obstetric outcomes – higher risk in patients with endometriosis					
**Higher quality studies**	**POPULATION**	**ENDOMETRIOSIS DIAGNOSIS**	**MODE OF CONCEPTION**	**WAS FIV TAKEN AS A BIAS?**	**STAGE OF THE DISEASE TAKEN AS ACCOUNT?**
Berlac et al, 2017^16^	11,739 women with endometriosis diagnosis vs. 615,533 women without endometriosis diagnosis.	Clinical ICD-10	Natural and artificial	Yes	Yes
Glavind et al, 2017^17^	82,793 women, 1,719 cases with endometriosis diagnosis.	ICD 10 and/or laparoscopic	Natural and artificial	Yes	No
Mannini et al, 2016^18^	Cases n= 262. A) 40 with DIE B) 222 without DIE (B.1- 188 singleton pregnancy and/or spontaneous pregnancy / B.2- 74 multiple gestation and/or FIV) vs. Control *n* = 524 without endometriosis.	Surgical and anatomopathological	Natural and artificial	Yes	Yes
Saraswat et al, 2016^19^	Cases *n* = 5,375 women with endometriosis diagnosis vs. Control *n* = 8280 women without endometriosis.	Surgical	Not evaluated	No	No
Stephansson et al, 2009^20^	Cases (*n* = 13,090 endometriosis diagnosis) vs. Control (*n*= 1,429,585)	Clinical ICD-8: 625.3; ICD-9: 617; and ICD-10: n80	Natural and artificial	No	No
**Lower quality studies**	**POPULATION**	**ENDOMETRIOSIS DIAGNOSIS**	**MODE OF CONCEPTION**	**WAS FIV TAKEN AS A BIAS?**	**DEGREE OF THE DISEASE TAKEN AS ACCOUNT?**
Turocy et al, 2017^21^	Women with transferred embryos (*n* = 1,616), 160 in the n with diagnosis of endometriosis.	Surgical	Artificial	No	Yes
Santulli et al, 2016^22^	Case *n* = 284, women diagnosed with endometriosis (A. superficial 52 / B. endometrioma / C. Deep infiltration) vs. Control *n* = 466, women without diagnosis of endometriosis.	Surgical and anatomopathological	Natural and artificial	Yes	Yes
Fujii et al, 2016^23^	Case *n*= 92 women with endometriosis diagnosis vs. Control *n*= 512 women without endometriosis	Laparoscopic	Artificial	No	Yes
Jaques et al, 2016^6^	2,316 pregnancies by assisted reproduction and 160 with diagnosis of endometriosis	Surgical or clinical by image + clinical exam	Artificial	No	Yes
Lin et al, 2015^24^	249 cases (women with endometriosis) vs. 249 controls (women without endometriosis)	Surgical and anatomopathological	Natural	No	No
Conti et al, 2014^25^	Population 2,239 women. Singleton pregnancy 1,331 control vs. 219 cases diagnosed with endometriosis. Multiparas: 592 control vs. 97 with diagnosis of endometriosis.	Surgical and anatomopathological	Natural and artificial	Yes	Yes
Aris, 2014^26^	Cases *n* = 784 women with endometriosis vs. Control *n* = 30,284 women without endometriosis.	Surgical	Natural and artificial	No	No
Mekaru et al, 2013^27^	108 pregnant women who had previously undergone laparoscopy to investigate infertility. 49 cases diagnosed with endometriosis vs. 59 controls.	Laparoscopic	Natural	No	Yes
Vercellini et al, 2012^28^	419 cases (150 rectovaginal, 69 ovarian and peritoneal, 100 ovarian, 100 peritoneal)	Clinical by image	Natural	No	Yes
Hadfield et al, 2009^29^	Cases (*n*= 3,239 with endometriosis diagnosis) vs. Control (*n* = 205,640)	Clinical ICD-10	Natural and artificial	Yes	Yes
Brosens et al, 2007^30^	Cases (*n* = 245 with diagnosis of infertility associated with endometriosis) vs. Control (*n* = 274 infertility associated with male factors)	Laparoscopic	Artificial	No	No
Hjordt Hansen et al, 2007^31^	Cases n= 24,667 women with endometriosis diagnosis vs. Control *n*= 98,668 women without endometriosis.	Clinical - ICD10 80–80.9	Natural or artificial	No	Yes
Matorras et al, 1998^32^	Cases *n* = 174 infertile women diagnosed with endometriosis. Control *n* = 174 infertile women without endometriosis.	Laparoscopic	Natural or artificial	Yes	Yes

Abbreviations: DIE, Deep infiltrative endometriosis; FIV, in vitro fertilization.

**Table 2a:** – Evaluation of the following risks of adverse obstetric outcomes: abortion, ectopic gestation, congenital malformations, fetal loss, stillborn and neonatal death in women with endometriosis according to the quality of the studies analyzed.

Obstetric outcomes – higher risk in patients with endometriosis						
**Higher quality Studies**	**Abortion**	**Ectopic gestation**	**Congenital malformation**	**Fetal loss**	**Stillborn**	**Neonatal death**
Berlac et al, 2017^16^			OR 1.3; 95%CI 1.3–-1.4			OR 1.8, 95%CI 1.4–2.1
Glavind et al, 2017^17^						
Saraswat et al, 2017^19^	OR 1.76; 95%CI 1.44–-2.15	OR 2,70; 95%CI 1.09–-6.72				NR
Mannini et al, 2016^18^						
Stephansson et al, 2009^20^						
**Lower quality Studies**	**Abortion**	**Ectopic gestation**	**Congenital malformation**	**Fetal loss**	**Stillborn**	**Neonatal death**
Turocy et al, 2017^21^	OR 0.57; 95%CI 0.28–1.15	OR 1.52, 95%CI 0.19–11.93				
Santulli et al, 2016^22^	OR 1.70, 95%CI 1.34–2.26					
Fujii et al, 2016^23^						
Jaques et al, 2016^6^						
Lin et al, 2015^24^						
Conti et al, 2014^25^						
Aris. 2014^26^	OR 1.89; 95%CI 1,23–2,93			OR 2.03; 95%CI 1,42–2,90	OR 2.29; 95%CI 1.24–5.22	
Mekaru et al, 2013^27^	NR					
Vercellini et al, 2012^28^						
Hadfield et al, 2009^29^						
Bronsens et al, 2007^30^						
Hjordt Hansen et al, 2007^31^	OR 1.2; 95% CI 1.2-1.3	OR 1.9, 95%CI 1.8–-2.1				
Matorras et al, 1998^32^	NR					

Abbreviation: NR, not relevant.

**Table 2b:** Evaluation of the following risks of adverse obstetric outcomes: pre-eclampsia, gestational diabetes, cholestasis, premature placental abruption, premature rupture of membranes and preterm labor in women with endometriosis according to the quality of the studies analyzed.

**Higher quality studies**	**Pre-eclampsia**	**Gestational diabetes**	**Cholestasis**	**Premature placental abruption**	**Premature rupture of membranes**	**Preterm labor**
Berlac et al, 2017^16^	OR 1.7; 95% CI 1.5–2.0			OR 2.0; 95% CI 1.7-2.3	OR 1.7; 95% CI 1.5-1.8	
Glavind et al, 2017^17^	OR 1.37; 95%CI 1.06–1.77					
Saraswat et al, 2017^19^	NR			NR		
Mannini et al, 2016^18^	NR	NR	OR 0.21; 95%CI 0.08–0.54			OR 0.32; 95%CI 0.20–0.52
Stephansson et al, 2009^20^						
**Lower quality studies**	**Pre-eclampsia**	**Gestational diabetes**	**Cholestasis**	**Premature placental abruption**	**Premature rupture of membranes**	**Preterm labor**
Turocy et al, 2017^21^						
Santulli et al, 2016^22^						
Fujii et al, 2016^23^						
Jaques et al, 2016^6^	OR 8.53; 95%CI 1.05–69.40					
Lin et al, 2015^24^	NR			NR		
Conti et al, 2014^25^	NR	OR 2.13; 95%CI 1.32–3.44			OR 2.93; 95%CI 1.24–6.87	
Aris. 2014^26^	NR	NR				
Mekaru et al, 2013^27^	NR					
Vercellini et al, 2012^28^						
Hadfield et al, 2009^29^	NR					
Bronsens et al, 2007^30^	OR 6.6; 95% CI 1.2–37					
Hjordt Hansen et al, 2007^31^						
Matorras et al, 1998^32^						

Abbreviation: NR, not relevant.

**Table 2c:** Evaluation of the following risks of adverse obstetric outcomes: bleeding during pregnancy, placenta previa, preterm newborn, placental retention, cesarean section, fetus small for gestational age and postpartum hemorrhage in women with endometriosis according to the quality of the studies analyzed.

**Higher quality studies**	**Bleeding during pregnancy**	**Placenta previa**	**Pre term newborn**	**Placental retention**	**Cesarian**	**Small for gestacional age**	**Postpartum haemorrhage**
Berlac et al, 2017^16^	OR 2.3; 95%CI 2.0–2.5	OR 3,9; 95%CI 3.5–4.3	OR 3.1; ,95%CI 2.7–3.6	OR 3.1, 95%CI 1.4–6.6		OR 1.5; 95%CI 1.4–1.6	
Glavind et al, 2017^17^	NR		OR 1.91; 95%CI 1.16–3.15		OR 1.83; 95%CI 1.60–2.09	NR	
Saraswat et al, 2017^19^	OR 1.67; 95%CI 1.39–2.0	OR 2.24; 95%CI 1,52-3.31	OR 1.26; 95%CI 1.07–1.49		OR 1.4; 95%IC 1.26–1.55	NR	OR 1.30; 95%CI 1.61–1.46
Mannini et al, 2016^18^	NR	OR 0.29; 95%CI 0.10–0.81			NR		
Stephansson et al, 2009^20^			OR 1.33; 95%CI 1.23–1.44				
**Lower quality studies**	**Bleeding during pregnancy**	**Placenta previa**	**Pre term newborn**	**Placental retention**	**Cesarian**	**Small for gestational age**	
Turocy et al, 2017^21^							
Santulli et al, 2016^22^							
Fujii et al, 2016^23^		OR 15.1,;95% CI 4,40–61.7	OR 2.08, 95%CI 1.07–3.89				
Jaques et al, 2016^6^	OR 2.05; 95%CI 1.02–4.11		OR 2.34; 95%CI 1.01–5.41		OR 2.64; 95%CI 1.37–5.07		
Lin et al, 2015^24^		OR 4.51; 95%CI 1.23–16.50	OR 2.42; 95%CI 1,05–5,57		OR 1.93; 95%CI 1.31–2.84	NR	
Conti et al, 2014^25^			OR 2.24; 95%CI 1.46–3.44			OR 2.72; 95%CI 1.46-5.06	
Aris et al, 2014^26^						NR	
Mekaru et al, 2013^27^					NR	NR	
Vercellini et al, 2012^28^		OR 5.81; 95%CI 1.53–22.03					
Hadfield et al, 2009^29^							
Bronsens et al, 2007^30^							
Hjordt Hansen et al, 2007^31^							
Matorras et al, 1998^32^							

Abbreviation: NR, not relevant.

Obstetric outcomes – higher risk in patients with endometriosis.

## Summary of the Studies with Superior Quality of Evidence

The largest and most detailed publication for the assessment of obstetric and neonatal complications in women with endometriosis was published in 2017 by Berlac et al.^16^ In this retrospective cohort study, data from every pregnant woman registered in Denmark at The National Health Register were computed from women with clinically diagnosed endometriosis. They were identified as having been diagnosed through the ICD-10 classification and were compared with women without the disease.^16^

The study performed by Berlac et al^16^ is listed in Table 2.

A sub-analysis was also performed for primiparous women and those who underwent gynecological surgery before pregnancy, as listed in Table 1.

These data is all shown in Tables 2a, 2b and 2c and are described here as follows. Berlac et al^16^ found in a cohort of 19,331 deliveries (case group with 11,739 women and control group with 6,533 women) increased risks in the group diagnosed with endometriosis for: pre-eclampsia (OR 1.7; 95% CI 1.5–2.0), bleeding during pregnancy (OR 2.3; 95% CI 2.0–2.5), premature placental abruption (OR 2.0; 95% CI 1.7–2.3), placenta previa (OR 3.9; 95% CI 3.5–4.3), premature rupture of membranes (RPMO) (OR 1.7; 95% CI 1.5–1.8), placental retention (OR 3.1; 95% CI 1.4–6.6), preterm newborn with < 28 weeks (OR 3,1; 95% CI 2.7–3.6), SGA (OR 1.5; 95% CI 1.4–1.6), congenital malformation (OR 1.3; 95% 1.3–1.4), neonatal death (OR 1.8; 95% CI 1.4–2.1).^16^

Glavind et al^17^ also conducted a large retrospective cohort study to examine the association between endometriosis and the risk of pre-eclampsia, cesarean delivery, postpartum hemorrhage, preterm delivery, and birth to SGA infants. The data were obtained from the Aarhus birth Cohort, a Danish national registry of patients of 82,793 of one-fetus pregnancies. Of these, 1,213 were diagnosed with endometriosis and 1,719 pregnancies were included in the group of women to be studied.^17^

The diagnosis of endometriosis was validated based on the identification of ICD 10–N80 and ICD 8–625.3 taken from the national database of patients. The results corroborated with laparoscopic confirmation in 33% of the cases; however, the severity of the disease was not taken into account. This was the first large study with histopathological confirmation of the diagnosis of endometriosis. The results are presented in Table 2c. An increased risk of preterm birth (OR 1.91; 95% CI 1.16–3.15), pre-eclampsia (OR 1.37; 95% CI 1.06–1.77) and delivery by cesarean section (OR 1.83; 95% CI 1.60–2.09) was found. There was no association with postpartum hemorrhage or SGA.^17^

In 2017, Mannini et al^18^ conducted a retrospective cohort at a tertiary hospital in Berlin between January 2009 and December 2014. The case group contained 262 pregnant women with surgical diagnosis of endometriosis, and 524 women without this disease in the control group. Results are shown in Tables 2b and 2c. Increased risk was shown in patients with endometriosis for placenta previa (OR 0.29; 95% CI 0.10–0.81), intrahepatic cholestasis (OR 0.21; 95% CI 0.08–0.54), labor induction (OR 0.05; 95% CI 0.34–0.69) and preterm delivery (OR 0.32, 95% CI 0.20–0.52). There was no association with transient hypertensive gestational disease, gestational diabetes, hemorrhage, cesarean delivery or intrauterine growth restriction.^18^

From 1981 to 2010, a cohort study evaluated data from all Scottish hospitals as reported by Saraswat et al.^19^ A total of 42,092 women were diagnosed with endometriosis and 8,719 women were identified as having had postdiagnosis pregnancies. Women without surgical diagnosis (*n* = 2,962) were excluded from the case group, as the author only included patients who had confirmed the disease through laparoscopy (98.7%) or laparotomy (1.3%). Results are summarized in Table 2. The authors reported increased risk in women with the diagnosis of endometriosis for abortion (OR 1.76; 95% CI 1.44–2.15), ectopic pregnancy (OR 2.70; 95% CI 1.09–6.72), placenta previa (OR 2.24; 95% CI 1.52–3.31), antepartum hemorrhage (OR 1.67; 95%, CI 1.39–2.0), postpartum hemorrhage (OR 1.30; 95% CI 1.61–1.46), preterm birth (OR 1.26, 95% CI 1.07–1.49) and cesarean delivery (OR 1.4; 95% CI 1.26–1.55). There was no association with transitory hypertensive disease during pregnancy, pre-eclampsia, placental abruption, SGA and stillbirth.^19^

Stephansson et al^20^ published in 2009 a large retrospective study that examined the association between unfavorable obstetric outcomes, assisted reproduction and endometriosis. The data were taken from the medical birth register, a database of the Swedish population, between the years 1992 and 2006. The case group included 13,090 one-fetus pregnancies of women diagnosed with endometriosis. As a result see Tables 2a, 2b and 2c; there was an increased risk for preterm birth (OR 1.33; 95% CI 1.23–1.44), pre-eclampsia (OR 1.13; 95% CI 1.02–1.26), antenatal bleeding and placental complications (OR 1.76; 95% CI 1.56–1.99) and cesarean delivery (OR 1.47; 95% CI 1.54–1.75).^20^

## Summary of Studies with Moderate Quality of Evidence

Studies that assessed smaller control groups than those mentioned above also showed a correlation between unfavorable obstetric outcomes and women diagnosed with endometriosis. The oldest of them evaluated 174 women with endometriosis and compared it to the same number of women without diagnosis.^32^ The authors examined the possibility of higher rates of abortion in the case group, but did not observe this correlation. Mekaru et al^27^ also reached this result after evaluating a group of 108 pregnant women who had previously undergone laparoscopy to investigate infertility. In contrast, Saraswat et al,^19^ Turocy et al,^21^ Santulli et al,^22^ Aris,^26^ and Hjordt Hansen et al^31^ published results showing increased risk of abortion in women with endometriosis.

When evaluating the correlation between transient hypertensive disease during pregnancy and endometriosis, most studies did not report this association, as shown in the studies by Mannini et al,^18^ Saraswat et al,^19^ Lin et al,^24^ Conti et al,^25^ Aris,^26^ Mekaru et al,^27^ Hadfield et al,^29^ and Brosens et al.^30^ While specifying the obstetric outcome for pre-eclampsia, some results did show statistical significance when related to the diagnosis of endometriosis, as described by Berlac et al,^16^ Glavind et al^17^ and Brosens et al.^30^ But the results reported by Saraswat et al,^19^ Conti et al^25^ and Aris^26^ disagreed as they show negative association between endometriosis and the outcome in question.^9^ ^16^ ^17^ ^18^ ^19^ ^24^ ^25^ ^26^ ^27^ ^29^ ^30^

Many authors also evaluated the relationship between pregnant women with endometriosis and placental disorders such as premature rupture of membranes, placenta previa and premature placental abruption. Berlac et al,^16^ Conti et al,^25^ and Harada et al,^33^ who included premature rupture of membranes in the studied outcomes, concluded that women with endometriosis are a risk group to present these pathologies. The investigated placental outcomes were linked to the diagnosis of endometriosis in the studies by Berlac et al,^16^ Mannini et al,^18^ Saraswat et al,^19^ Fujii et al,^23^ Lin et al^24^ and Vercellini et al^28^ and Harada et al,^33^ showing a strong correlation among them. Premature placental abruption was identified to be associated with endometriosis in the studies by Berlac et al^16^ and Harada et al.^33^ However, Lin et al^24^ did not find the same association.^16^ ^18^ ^19^ ^23^ ^24^ ^25^ ^28^ ^29^ ^30^ ^31^ ^32^ ^33^ ^34^

Many authors place newborn-related outcomes among the unfavorable obstetric results to be evaluated in women with endometriosis. All of the studies that evaluated preterm birth found a positive correlation, as reported by Berlac et al,^16^ Glavind et al,^17^ Saraswat et al,^19^ Stephansson et al,^20^ Fujii et al,^23^ Jacques et al,^34^ Lin et al,^24^ and Conti et al.^25^ Berlac et al^16^ and Conti et al^25^ included SGA fetuses, and also obtained a positive correlation with increased risk in women with endometriosis.

The delivery route was also evaluated in many studies included in the present systematic review. Glavind et al,^17^ Saraswat et al,^19^ Jacques et al^34^ and Lin et al^24^ reported higher risk of cesarean delivery in women diagnosed with endometriosis. However, Mannini et al^18^ and Mekaru et al^27^ did not report this association.^17^ ^19^ ^24^ ^27^ ^34^

## Discussion

The present systematic review highlighted observational studies, some more robust with larger control groups and others with more restricted groups (Table 1). The highest agreement between studies of greater quality of evidence is the high incidence of preterm birth and placenta previa in patients with endometriosis.^16^ ^18^ ^19^ Moderate quality studies also showed endometriosis-diagnosed patients to have more abortion occurrences and cesarean deliveries.^21^ ^22^ ^24^ ^25^ ^31^ ^34^ Generally, most studies highlight the impact of endometriosis on unfavorable obstetric outcomes, although only three less relevant case-control group studies found no evidence of higher risk (Table 2).^27^ ^29^ ^32^

The causes of higher risk for obstetric complications have not yet been defined, and the underlying pathophysiological factors are still unclear.^35^ Despite this, endomyometrial changes present in patients with endometriosis seem to be responsible for several obstetric adverse factors such as abortion, fetal growth restriction, placenta previa, and preterm delivery or SGA infants.^35^ More specifically, in relation to the increased incidence of complications such as preterm birth and placenta previa, we can emphasize alterations in endometrial hormonal receptivity, decidualization and remodeling of the spiral uterine arteries and inflammatory state that alter the regulation of the endocrine immune system in patients with endometriosis.^18^ ^36^

The reason why some placentas are implanted in the lower segment of the uterus remains under discussion. As gestation progresses, 90% of low-insertion placentas move towards the uterine fundus. The placenta grows preferably towards the best-vascularized area, which is the uterine fundus (trophotropism), and the placenta that remains in the least vascularized area undergoes atrophy. Uterine contractions lead to detachment of this area of the placenta and subsequent bleeding, which further stimulates uterine contraction.^37^ Resistance to progesterone and inadequate uterine contractions occurring in women with endometriosis may explain the greater frequency of placenta previa in this subgroup.^18^ Endometriosis also leads to a hyperinflammatory state of the endometrium that causes endometrial endocrine immune balance disorder (increase in sex hormones, neurohormones, cytokines and growth factors). This disorder is thought to influence the interaction of decidua/trophoblast and activate the mechanisms of preterm delivery when the imbalance between pro-inflammatory and anti-inflammatory mechanisms of the placenta occurs.^7^ These mechanisms found in patients with endometriosis may justify the greater obstetric risks.

In the last 20 years, several studies focused on the evaluation of this diversity of obstetric complications, with premature rupture of membranes and cases of placenta previa being the most commonly associated complications in the representative studies. Reviews on the topic report the same aforementioned complications in women diagnosed with endometriosis as those found in the present systematic review. However, in our review, we perceived that among all analyzed studies, there is great statistical heterogeneity and their quality was not taken into account. Therefore, to be able to derive significant conclusions from our study we chose to assign greater importance to the quality of each of the studies. This approach minimized the possible biases of each study and allowed a better analysis of the results.^13^

At the same time, there is a concern in the literature regarding the results found in a number of different studies (bias, false positives or false negatives).^38^ ^39^ In this respect, systematic reviews are able to extract from the studies information on data quality, sample size, possible biases and methodological description. Despite the volume of studies published with endometriosis in pregnancy, there is the need to prepare large studies, with carefully selected control groups (to avoid bias), based on a working hypothesis compatible with existing results from previous reviews, and focusing on the association and risk between endometriosis and unfavorable obstetric outcomes.

Nonetheless, our study also presents limitations in view of those of each of the articles analyzed. Inherent characteristics of many studies, such as methodological flaws, lack of histological confirmation of endometriosis, and small number of patients were a frequent finding.^16^ ^32^ Another divergence among the studies was whether infertility and in vitro fertilization were considered as a bias (Table 1). It is important to note that endometriosis and infertility may be independent risk factors, since polymorphisms in genes associated with infertility, regardless of endometriosis, are also related to unfavorable obstetric outcomes.^40^ To minimize the mentioned limitations of many studies, we used the NOS scale method to classify and assign higher or lower quality to each study as explained.

Finally, through the present data compilation, it is possible to direct the search for endometriosis-associated obstetric complications looking for the underlying causes and mechanisms. It also informs on possible guidelines for the clinical care of patients with surgical diagnosis of endometriosis, in order to reduce the rates of comorbidities associated with endometriosis in pregnancy.

## Conclusion

Endometriosis is a disease that extends beyond the presence of ectopic endometrial implants. The condition of the endometrium can determine the quality of implantation and placental development, influencing obstetric outcomes, especially preterm birth and placenta previa. More studies paying more attention to the quality of the methodology, with adequate experimental designs and without bias such as in vitro fertilization, are necessary. The information gathered is useful to alert obstetricians and women diagnosed with endometriosis about possible unfavorable obstetric outcomes.
