# Supplementary material for: Is there an Increased Risk for Unfavorable Obstetric Outcomes in Women with Endometriosis? An Evaluation of Evidences
Source: Rev Bras Ginecol Obstet. 2020 Apr;42(4):200–10. doi: 10.1055/s-0040-1708885 (PMC10316868; doi:10.1055/s-0040-1708885)
Supplement: Supplementary file 1 — Supplementary Material [file 10-1055-s-0040-1708885-s190260.pdf]

Supplementary Table 1 Newcastle–Ottawa Scale (NOS) for the selection of study groups

QUALITY ASSESMENT	SELECTION	COMPARABILITY	OUTCOME/EXPOSURE	TOTAL
Berlac et al. 2017[16]	****	**	***	9
Glavind et al. 2017[17]	****	**	***	9
Mannini et al. 2016[18]	****	**	***	9
Stephansson et al. 2009[20]	****	*	***	8
Saraswat et al. 2016[19]	****	*	***	8
Turocy et al. 2017[21]	***	*	**	6
Santulli et al. 2016[22]	***	**	*	6
Fujii et al. 2016[23]	**	*	***	6
Jacques et al. 2016[6]	**	*	**	5
Lin et al. 2015[25]	***	*	***	7
Conti et al. 2014[26]	***	**	**	7
Aris et al. 2014[27]	***	*	*	5
Mekaru et al. 2013[28]	**	*	*	4
Vercellini et al. 2012[29]	**	*	***	6
Hadfield et al. 2009[30]	***	**	*	6
Brosens et al. 2007[31]	***	*	*	5
Hjordt et al. 2007[32]	***	*	**	6
Matorras et al. 1998[33]	***	**	*	6
